# Safety of Off-Label Pharmacological Treatment in Pediatric Neuropsychiatric Disorders: A Global Perspective From an Observational Study at an Italian Third Level Children’s Hospital

**DOI:** 10.3389/fphar.2022.837692

**Published:** 2022-04-12

**Authors:** Maria Sole Giurin, Marta Paulina Trojniak, Anna Arbo, Marco Carrozzi, Giuseppe Abbracciavento, Lorenzo Monasta, Caterina Zanus

**Affiliations:** Institute for Maternal and Child Health - IRCCS “Burlo Garofolo”, Trieste, Italy

**Keywords:** off-label, antipsychotics, antiepileptics, pediatric, pharmacovigilance, clinical pharmacist, safety, neuropsychiatric disorders

## Abstract

**Background:** The acquisition of proper and relevant pediatric clinical data is essential to ensure tolerable and effective pediatric drug therapies. In the field of pharmacological treatment of neuropsychiatric disorders, the lack of sufficient high quality scientific evidence for pediatric age results in the frequent need to prescribe off-label drugs. With the aim of improving knowledge about safety profile of off-label drug prescription in children and adolescent with neurological and/or psychiatric disorders, we realized a multidisciplinary pharmacovigilance study.

**Materials and methods:** An observational retrospective study was conducted to assess the safety of off-label pharmacological therapies in patients aged 0–18 years, admitted to the Neuropsychiatry Unit of the Institute for Maternal and Child Health - IRCCS “Burlo Garofolo” between January 2016 and December 2018. Prescription patterns and adverse drug reactions were evaluated by a multidisciplinary team.

**Results:** Overall, 230 patients were enrolled, 48% boys (N = 111), 52% girls (N = 119), average age of 10 years, and a total of 534 prescriptions was analyzed. 54.5% (N = 125) of patients had epilepsy, 37.5% (N = 86) suffered from psychiatric disorders, 8% (N = 19) had other neurological disorders. The prevalence of off-label prescriptions was 32% and 50% of the study population received at least one off-label drug. A total of 106 ADRs was detected: 57% of ADRs were due to drug-drug interactions, 30% were due to off-label prescriptions, 10% were due to overdose and 3% were due to improper use. No significant association between emerged ADRs and off label prescriptions was found (Fisher’s exact two-tailed test, *p* = 1.000). There was significant association between increasing number of administrated drugs and risk of ADRs (OR 1.99; IC95% 1.58–2.5; *p* = 0.000). Psychiatric disorders were associated with at least three times higher risk to be treated with an off-label drug (OR 3.30; IC95% 2.26–4.83; *p* = 0.000).

**Conclusions:** This study shows that off-label prescribing in neuropsychiatric disorders does not pose a greater risk of ADRs than on-label prescribing and highlights unmet clinical needs in pediatric neuropsychopharmacology. The multidisciplinary approach can provide important contributions to improve therapeutic path of these already complex pathologies by careful monitoring of therapeutic appropriateness and drug interactions.

## Introduction

Children are not little adults, especially when they take drugs, not only do they differ from adults in size, but also as far as drugs’ absorption, metabolism and excretion are concerned (Wagner and Abdel-Rahman 2013).

Due to ethical and commercial problems and to the lack of pharmaceutical research on pediatric neurology and psychiatry, some medications are unlicensed, holding no marketing authorization, or are used outside the indication or age range for which they are licensed. This makes selecting an appropriate drug even more complex. Pediatric neurology and psychiatry are therapeutic areas frequently requiring off-label approaches and this practice is challenging for prescribing physicians. Beside authorized indications, some off-label uses with good clinical evidence are already authorized and reimbursed in Italy based on Law 648/1996. Tables 1 and 2 show authorized and reimbursed indications of antiepileptic and psychotropic drugs for children and adolescents in Italy. However, the limited indications of these drugs lead to widespread use of off-label prescriptions.

**TABLE 1:** Authorized and reimbursed indications of antiepileptic drugs for Children and Adolescents in Italy.

Drug name	Pediatric approved indications	Off label use authorized by law 648/96
ACTH	Infantile Epileptic, Encephalopathy with hypsarrhythmia	Add-on: ESES, Lennox-Gastaut syndrome, Severe Epileptic Encephalopathy
Ethosuximide	Absence Epilepsy	Add-on: ESES, Epileptic Negative Myoclonus
Lamotrigine	2–12 years:	Monotherapy for the treatment of Janz syndrome in >12 years old
	- Monotherapy for typical absences	
	- in add-on in focal seizures, generalized TC and Lennox-Gastaut syndrome	
Levetiracetam	Focal seizures with or without secondary generalization:	Monotherapy >12 years for the treatment of Jan syndrome, ESES, Add-on: Typical absences
	- monotherapy: > 16 years old	
	- Add on: > 1 month	
	- 12 years Janz syndrome	
Rufinamide	Add-on: Lennox-Gastaut syndrome >4 years	Add-on for severe Encephalopathy >4 years
Topiramate	Focal and generalized seizures:	Drug-resistant Typical absence seizures
	- monotherapy: > 6 years	
	Lennox-Gastaut syndrome	
	- Add-on> 2 years	
Zonisamide	No pediatric indications	Severe epileptic encephalopathies >4 years in add-on Typical pharmacoresistant absences
Clobazam	No indications in epilepsy	Severe drug resistant epilepsies over 3 years old
Dexamethasone	No indications in epilepsy	Not present in L. 648/96
Nitrazepam	No indications in epilepsy	Not present in L. 648/96
Perampanel	In add-on in patients aged> 12 years for the treatment of focal seizures, generalized TC	Not present in L. 648/96

**TABLE 2:** Authorized and reimbursed indications of antipsychotic drugs in Children and Adolescents in Italy.

Drug name	Pediatric approved indications	Off label use authorized by law 648/96
Risperidone	Indicated for persistent aggressiveness associated to conduct disorder in children (>5 years old) and adolescents with intellectual disabilities or limit intellectual functioning, diagnosed according to DSM-V criteria	- Short-term treatment of moderate or severe behavioral problems such as irritability and aggression in individuals (≥5 years) with autism spectrum disorders
		- Tourette syndrome with moderate to severe functional impairment (≥7 years)
		- Add-on to methylphenidate in subjects (≥7 years old) with ADHD and oppositional defiant disorder, or aggressive behavior who have not responded effectively to methylphenidate treatment alone
Olanzapine	No pediatric authorization	>7 years schizophrenia and bipolar disorder
Quetiapine	No pediatric authorization	>12 years schizophrenia and bipolar disorder
Aripiprazole	>15 years schizophrenia	>13 years schizophrenia
	>13 years bipolar I disorder	>10 years type1 bipolar disorder
		>6 years treatment of irritability in subjects with autism spectrum disorders
		>6 years Tourette’s syndrome
Clozapine	>16 years schizophrenia and psychosis	Acute and chronic psychosis in adolescents and children
		>7 years of age
Delorazepam	No pediatric authorization	Not present in L. 648/96
Clothiapine	No pediatric authorization	Not present in L. 648/96
Promazine	Patients older than 12 years	Not present in L. 648/96
	- Treatment of psychomotor agitation or aggressive behavior	
	- Schizophrenia and other psychotic disorders	
Lithium	Prophylaxis and treatment of	Not present in L. 648/96
	- states of excitement in forms of mania and hypomania	
	- states of depression or chronic depressive psychosis manic-depressive psychosis	
Fluoxetine	>8 years major depression	Not present in L. 648/96
Sertraline	6 years obsessive compulsive disorder	Not present in L. 648/96

The Italian legislation governing the use of off-label drugs refers to Law 94/1998 (Law N 94/1998, 1998). The term “off-label” refers to an authorized pharmaceutical product used outside the terms of its marketing authorization and consequently not in line with the information contained within the summary of its characteristics.

The lack of clinical trials in pediatric population involves uncertainties in terms of efficacy and safety, this means that evidence derived from real world is crucial to define the benefit-risk profile of drugs in the pediatric population.

It is known that children are more subject to adverse drug reactions (ADRs) than adults, so the use of off-label drugs may expose them at a higher risk of toxicity or ineffective treatment (Rosli et al., 2017). There is little evidence about the use and the risk of ADRs associated with off-label drugs in pediatric neuropsychopharmacology, and scientific communities recognize the urgent need to carry out epidemiological studies not only on the frequency of off-label prescriptions, but also on the indications for their use and the monitoring of these therapies (Sharma et al., 2016).

In spite of this uncertainty, off-label prescribing is a tool of early access, particularly widespread in pediatrics, which allows physicians to treat young patients.

Pharmacists and pharmacologists play a significant role in the drug monitoring, consisting in appropriateness evaluation through a systematic review of the scientific literature, with the aim of identifying and summarizing evidence on the effectiveness and safety of the pharmacologic interventions. Pharmacotherapy is pivotal in treating patients with psychiatric and neurologic disorders; however, its success is often limited by adverse effects, inefficacy, inadequate therapy monitoring and follow up, and poor adherence (Persico et al., 2015). In many cases, adverse effects go unrecognized. The non-identification of ADRs can have dire consequences, leading to the so-called “prescription cascade”. A lot can be done by the multidisciplinary team to prevent side effects, mainly: monitoring of therapies, patient education (advise the patient on how to best take his or her medications to maximize benefits while minimizing side effects), follow-up, critical appraisal of the literature, polypharmacy review and drug-drug interactions, develop a treatment plan to resolve any medication-related problems (e.g. change administration times; propose therapeutic drug monitoring and change dose if the therapy reveals to be toxic or ineffective, prepare information documents to inform the patient about the therapy and side effects so that he/she can recognize them early) (Werremeyer et al., 2020).

The lack of sufficient high quality scientific evidence and the frequent need to prescribe off-label drugs prompted us to start a pharmacovigilance study, with the aim of detecting ADRs and assessing safety profiles of off-label drug prescriptions as compared to on-label drugs.

With a multidisciplinary approach, the study was conceived through the collaboration of pharmacists, pharmacologists and clinicians who, at our Institute, routinely cooperate in all the prescription process.

## Materials and Methods

### Objectives

This study aimed to assess if off-label use in third level children’s hospitals may be associated with an increased risk of adverse drug reactions and to verify the real-world safety of these drugs.

Primary endpoint of the study:

‐ Estimate the incidence of adverse reactions in patients undergoing off-label drugs compared to the on-label in the inpatient setting of Pediatric Neuropsychiatry Department.

Secondary endpoints were:

‐ Analysis of the Prescriptions: estimate the prevalence of off-label drugs compared to on-label drugs, the frequency of different drugs used for off-label prescriptions and the indication for their use; analyze the quantity, quality, and consistency of evidence of off-label prescriptions; quantify the drug-drug interactions; evaluate the difference of off-label use between neurologic and psychiatric disorders.
‐ Analysis of the ADRs: estimate the prevalence of ADRs, characterization of ADRs and detection of ADR’s risk factors.

The study was performed with the approval of the regional ethics committee.

### Patients

To be eligible for participation in this study, patients had to be younger than 18 years; have confirmed evidence of psychiatric or neurological diagnosis; have been exposed to at least one medication and being hospitalized in the Pediatric Neuropsychiatry Department of the Institute for Maternal and Child Health - IRCCS “Burlo Garofolo” in Trieste from 2016 to 2018. In the Italian organization of medical services, Neuropsychiatry deals with all the neurological and psychiatric disorders of children and adolescents, different from other European countries where pediatric neurological and psychiatric domains are separated.

### Study Design

This is a retrospective, single-center, observational study.

The review of the cases was conducted through a multidisciplinary collaboration between physicians, pharmacists and pharmacologists with the aim of evaluating the appropriateness of the patients’ pharmacological treatment. For every single patient enrolled in the study the pharmacist analyzed and collected: socio-demographic information (age, gender, weight, height); diagnosis; drug prescriptions during hospitalization found in the medical records (duration of treatment, indication, dosage, time of administration, a route), exposure to off-label/on label drug, drug-drug interactions and safety analysis. All prescriptions during patients’ hospitalization were considered in the analysis.

Information regarding therapies and the clinical data (ADRs and biochemical parameters) of the study population was obtained from prescriptions in the electronic health record (clinical diary, drug prescription sheet), Hospital Mission Sheets (SDO), hospital discharge report and laboratory reports.

The collected clinical data was anonymized, and inserted into a certified database REDCap. The diagnoses were classified using the International Classification of Diseases, ninth revision (ICD - ICD-9-CM - International Classification of Diseases, 2021).

#### Classification of Medicines

The prescribed medicines were divided into: on-label (authorized and reimbursed indications including drugs form the list referred to the Law 648/1996) and off-label drugs (based on the Law 94/1998).

The evaluation was made by the pharmacist comparing the information from the prescriptions (patient’s age, diagnosis, etc.) with the information in SmPC (Summary of Product Characteristics) reported by AIFA (Italian Drug Agency) and EMA (European Medicines Agency). For each off-label prescription, scientific evidence available for the given off-label drug use was evaluated, mainly phase I, phase II, phase III studies, observational studies, case series and case reports.

#### Evaluation of Off-Label Prescriptions

The Scottish Intercollegiate Guidelines Network (SIGN) has been used to evaluate the quality of the evidence (Baird and Lawrence 2014). SIGN method leads to guidelines that are essentially the direct product critical appraisal of the systematic review.

The level of evidence depends on:

• Quantity, quality, and consistency of evidence;
• External validity (generalizability) of studies;
• Direct applicability of the guidelines to the target population.

#### Classification of Drug-Drug Interactions

Drug interactions were investigated by the pharmacist using three different data sources Lexicomp^®^ and Terap^®^ and SmPC.

Interactions were classified as:

• Avoid combination (X): when data demonstrate that the specified agent may interact with the other in a clinically significant manner. The risks associated with concomitant use of these agents usually outweigh the benefits. These agents are generally considered contraindicated;
• Consider therapy modification (D): when data demonstrate that the two medications may interact with each other in a clinically significant manner. A patient-specific assessment must be conducted to determine whether the benefits of concomitant therapy outweigh the risks. Specific actions must be taken in order to maximize the benefit and/or minimize the toxicity resulting from concomitant use of agent. These actions may include aggressive monitoring, empiric dosage changes, choosing alternative agents;
• Monitor Therapy (C): when data demonstrate that the specified agents may interact with each other in a clinically significant manner. The benefits of concomitant use of these medications usually outweigh the risks. An appropriate monitoring plan should be implemented to identify potential negative effects. Dosage adjustments of one or both agents may be needed in a minority of patients.
• No Action Needed (B): when data demonstrated that the specified agents may interact with each other, but there is little to no evidence of clinical concern resulting from their concomitant use.
• No known Interaction (A): data has not demonstrated either pharmacodynamic or pharmacokinetic interactions between the specified agents.

#### Classification of ADRs

The ADRs were coded using the Medical Dictionary for Regulatory Activities (MedDRA). ADRs were detected by pharmacists from electronic health records (clinical diary, drug prescription sheet), Hospital Mission Sheets (SDO), hospital discharge report and laboratory reports, and then confirm by physicians. For each ADR, the Summary of Product Characteristics (SmPC) was investigated by pharmacist to understand if the ADR was already reported and to understand its relevance. A “serious” ADR was defined as “any untoward medical occurrence that at any dose results in death, requires hospital admission or prolongation of existing hospital stay, results in persistent or significant disability/incapacity or is life threatening or other clinically relevant condition” (Gautron et al., 2018). To avoid correlation biases between the ADR and the drug, the Naranjo algorithm was calculated.

Furthermore, the causal link between ADRs and drug-drug interactions was assessed by pharmacist. The Drug Interaction Probability Scale (DIPS) algorithm was run to assess the causality between ADRs and drug-drug interaction.

Based on the result of the Naranjo algorithm and the DIPS algorithm, the ADRs related to the drug or interaction between drugs can be: doubtful (≤0); possible (1–4); probable (5–8); certain (≥9) (Horn et al., 2007).

### Statistical Analysis

For descriptive analyses, categorical variables were presented as frequencies and percentages, while continuous variables were reported as means and standard deviations (SDs) or as medians and interquartile ranges (IQR), as appropriate after verification of their distribution. The normality of the distribution of quantitative variables was tested by the Shapiro-Wilk test.

The main study outcome was the presence of ADRs, and the main independent variable was the prescription of off-labels versus on-labels. Associations were mainly studied using non-parametric tests. The two-tailed Fisher exact test was carried out to study the association between two dichotomous variables, while the Mann-Whitney rank-sum test was used to study the difference in the distribution of a continuous variables in two different groups.

Univariate and multivariate logistic regression have been used to study the association between type of drugs (off-label vs. on-label and drugs included in the Italian law 648/96) and health outcomes (ADRs, clinical outcomes), considering other relevant covariates that emerged in the study. Odds Ratios (ORs) and 95%CI were calculated for each variable included in the models.

The hypothesis of the study was to find a difference in the incidence of ADRs in the population of neuropsychiatric children undergoing off-label compared to on-label drugs of at least 20% (Bellis et al., 2014), based on our estimates of an ADR frequency of 10% in on-label and 30% in off-label uses. The total sample size needed was 200 subjects.

For all analyses, the significance level was set at p< 0.05. All analyses were performed using StataBE 17.0 (StataCorp, College Station, United States).

## Results

A total of 230 patients were enrolled: 48% boys (N = 111), 52% girls (N = 119), with an average age of 10 years. 54.5% (N = 125) of patients had epileptic diagnosis, 37.5% (N = 86) suffered from psychiatric pathologies, 8% (N = 19) had other neurological disorders.

### Primary Endpoint

There was no association between ADRs and the prescription of an off-label, among 71 prescriptions with at least one ADR detected, 49 of them (69%) were due to on-label prescriptions and 22 (31%) were related to off-label drugs (Table 3).

**TABLE 3:** Two-way relative frequency table between ADRs and off-label prescriptions.

		Off-label		Total	*p*-value of Fisher’s exact test
		No	Yes		
**Adverse Drug Reactions**	**No**	316	147	463	1.000
		68.3%	31.7%	100%	
		86.6%	87.0%	86.7%	
	**Yes**	49	22	71	
		69.0%	31.0%	100%	
		13.4%	13.0%	13.3%	
Total		365	169	534	
		68.4%	31.6%	100%	
		100%	100%	100%	

Considering the number of off-labels as a categorical variable, the Odds Ratio increases as the number of off-label administered increases. One off-label had an OR = 1.72 (95%CI 0.81–3.66; *p* = 0.1569), not significant. Two off-labels had an OR = 6.19 (95%CI 2,43–15.72; *p* = 0.000), while three off-labels had an OR = 15.47 (95%CI 2.76–86.62; *p* = 0.002). This analysis was not possible for the prescription of more than three off-label due to data scarcity.

### Secondary Endpoint

#### Analysis of the Prescriptions

A total of 534 prescriptions was analyzed. Off-label prescriptions represented 32% of the total (N = 169). 105 prescriptions referred to the psychiatric field, 52 to epileptic and 12 to other neurological problems.

In general, each patient received more than two therapies during hospitalization (Table 4). 50% of the patients (N = 115 out of 230) received at least one off-label drug with on average almost one and a half off-label prescriptions per person.

**TABLE 4:** Description of prescribing patterns.

Age groups	Freq	Off-label prescription	At least one off-label prescription	Average number of off-label prescriptions x person	Average number of on- and off-label prescription x person	Average number of drug-drug interaction per person
<1	17	10 (59%)	7 (41%)	1.57	2.29	1.00 (n = 3)
1–4	34	21 (62%)	13 (38%)	1.31	2.64	1.67 (n = 9)
5–11	60	36 (60%)	24 (40%)	1.42	2.35	1.68 (n = 19)
12–17	119	48 (40%)	71 (60%)	1.51	2.33	1.97 (n = 32)
Tot	230	115 (50%)	115 (50%)	1.47	2.32	1.79 (n = 63)

On the total of 534 prescriptions, 38 (7.2%) concerned medications included in the list based on Italian law 648/96.

Some 35.6% of off-label prescriptions were off-label with respect to the age of the patients, 44.4% with respect to the indication, 18.4% were both for age and indication in relation to the dose and 1.6% in relation to the therapeutic line.

The most common off-label drugs were: delorazepam, quetiapine, risperidone (off-labels with respect to age) and dexamethasone, nitrazepam, trihexyphenidyl (off-label with respect to indication).

The outcome related to the level of evidence and the grade of recommendation for off-label prescriptions result in 59.5% of grade 1A, 24.8% of grade 3D, 13.1% of grade 2C, 0.6% of grade 4D and 0.6% of grade 1B.

Sixty-three patients (27%) enrolled in the study experienced a drug-drug interactions (Table 4). A total of 113 drug-drug interactions were detected, with an average of 1.8 interactions per patient experiencing a drug-drug interaction. Of all drug-drug interactions, 59 were pharmacodynamic (52%), 45 were pharmacokinetic (40%), while nine were both (8%). Furthermore, 83% of drug-drug interactions were risk type C, requiring monitoring therapy and 17% were risk D, needing therapy modification. Valproic Acid, Carbamazepine, Lithium and Phenobarbital were the drugs causing most drug-drug interactions.

80% of patients with a psychiatric disorder have been exposed to more than one off-label drug. In the psychiatric field, off-label prescriptions are much more frequent: the analysis showed that psychiatric pathology is associated with a higher risk to be treated with an off-label drug (OR = 3.30, 95%CI 2.26–4.83; *p* = 0.000) if compared to other diagnoses.

There is also a significant association between drug-resistant epilepsy and prescription of off-label drugs (Table 5). 58% of the prescribed drugs to drug-resistant patients are off-label, while the percentage of prescribed off-label drugs to other patients is only 18%.

**TABLE 5:** Two-way relative frequency table between off-label prescriptions and drug-resistant epilepsy.

		Drug-resistant epilepsy		Total	*p*-value of Fisher’s exact two-tailed test
		No	Yes		
**Off-label**	**No**	72	16	88	0.000
		82%	18%	100%	
		82%	42%	70%	
	**Yes**	16	22	38	
		42%	58%	100%	
		18%	58%	30%	
Total		88	38	126	
		70%	30%	100%	
		100%	100%	100%	

Multinomial logistic regression has shown that the age group 12–17 years is the one with the highest risk of off-label prescriptions (RR 2.38, 95%CI 1.09–5.22; *p* = 0.029). The reference group was 1–4 years of age, the group with the lowest risk if compared to each of the others (RR 1.13, 95%CI 0.3–3.7; *p* = 0.8).

#### Analysis of the ADRs

During 71 drug administrations, at least one ADR was detected and the total of ADRs was 106, for an average of 1.5 per subject who had at least one.

Table 6 shows that 44% of all ADRs were neurological reactions, in particular extrapyramidal symptoms and dystonia (due to the use of first- and second-generation antipsychotics) and dizziness (caused by antiepileptic drugs). 18% were mental and behavioral disorders like mood alteration, insomnia and irritability (Table 6).

**TABLE 6:** Distribution of Adverse Drug Reactions reported by MedDRA System Organ Classes (SOCs).

Adverse drug reactions	Frequency	Percentage
SNC disorders	47	44
Mental and behavioral disorders	19	18
Gastrointestinal disorders	13	12
Immune system disorders	7	7
Cardiovascular disorders	6	6
Eyes disorders	3	3
Endocrine disorder	3	3
Urinary and renal disorders	3	3
Others disorders (reproductive, metabolism etc.)	5	5
Total Adverse Drug Reactions	106	100

Thirty-four medications were involved in adverse drug reactions: most ADRs were due to Risperidone (N = 17), Haloperidol (N = 9), Phenytoin (n = 8), Carbamazepine (N = 7) and Quetiapine (N = 6).

According to the Naranjo algorithm, 54% of all ADRs were probable, 35% possible, 7% certain and 4% doubtful. According to the DIPS algorithm, the causal link between ADRs and drug-drug interactions was 59% doubtful, 30% probable and 10% possible.

Thirty-four (32%) of a total of 106 ADRs were serious, one of them led to hospitalization or prolongation of hospitalization, the rest caused other clinically relevant conditions. Drugs that have caused serious adverse reactions were: Quetiapine, Valproic Acid, Risperidone, Phenytoin, Haloperidol, Vigabatrin, Dexamethasone, Oxcarbazepine, Levetiracetam, Clobazam. 65% of the ADRs caused serious CNS disturbances (drug-induced extrapyramidal adverse effect, convulsive seizure, hypotonia, dystonia and drowsiness), 3% caused CPK increase, 6% were related to reproductive and breast disease (gynecomastia and galactorrhea), 6% to endocrine disease (hyperprolactinemia, hypothyroidism), 3% gastrointestinal disease (mainly hypersalivation), 9% to cardiovascular disease (syncope hypotensive, tachycardia, retention water) and 9% to immune system disorders (tongue edema, rash and redness of face).

For some drugs, such as Haloperidol, Phenytoin, and Primidone, an ADR was observed in 50% of the administrations.

Overall, 57% of ADRs were due to drug-drug interactions, 30% to off-label prescriptions, 10% due to overdose and 3% due to improper use.

#### ADR’s Risk Factors

From the logistic regression analysis, it emerged that there were no significant associations between occurrence of adverse events and age group (OR 1.1; 95%CI 0.3–3.83; *p* = 0.17 for age range 12–17; the reference group was 1–4 years) and sex (OR 0.71; 95%CI 0.39–1.31; *p* = 0.28, for females). While there was a statistically significant association between increasing number of drugs taken by the patient and the onset of ADRs (OR 1.99; 95%CI 1.58–2.50; *p* = 0.000).

As regards to clinical risk management, 15 therapeutic errors and 14 near miss events were detected (3% and 3% of total prescriptions, respectively): 28 were due to medication errors (82% for drug-drug interactions of contraindicated drugs combinations and 29% for higher dose) and one due to administration errors (manipulation and administration error). It was calculated that the risk of an ADR is over six times higher if a prescription error occurs (OR 6.69, 95%CI 3.33–14.77; *p* = 0.000).

## Discussion

The primary endpoint of this study was to assess the risk of ADR associated with off-label prescribing in the field of pediatric neuropsychiatry. In our study, 30% of the ADRs involved an off-label prescription and ADR occurrence was not significantly related to off-label prescribing. Findings are consistent with several studies (Neubert et al., 2004; Mason et al., 2012; Palmaro et al., 2015) that found no association between ADR and off label prescribing. Other studies (Horen et al., 2002; Neubert et al., 2004; Aagaard and Hansen 2011; Bellis et al., 2014; Pratico et al., 2018) instead found significant association between ADR and off-label prescription. In these studies, ADRs were related mainly to anti-infective and anti-asthmatic drugs and vaccines, which were not prescribed in our study. Bellis et al. found an increased risk related to off label drugs used in oncological practice and the result became statistically insignificant when oncology patients were excluded (Bellis et al., 2014). Thus, it is possible that the greater risk of ADRs observed by the other studies resulted from a different pattern of off-label drug use (Rusz et al., 2021).

The fact that off-label prescribing in our pediatric sample with neurodevelopmental disorders shown not to be associated with ADRs leads us to focus more on finding evidence from the literature in order to provide the access to the pharmacological treatments offering the best possible care, regardless of marketing authorization.

This study highlights the unmet clinical needs of pediatric population with neurodevelopmental disorders: the frequent use of off-label prescriptions underlines the strong need for studies especially for psychiatric pathologies (OR = 3.30 for off-label prescription). The treatment of psychiatric disorders represents an important therapeutic challenge, determined by the complexity of the disorders and the difficulty of producing clinical pharmacological recommendations in the absence of consolidated evidence. Psychiatric patients are associated with a high risk of suicide, high access to mental health services, severe impairment of psychosocial functioning and high social and economic costs with the need for emergency hospitalization therefore the implementation of research in this field is very important (Leichsenring et al., 2011).

Considering the indications approved for neuroleptics drugs, it is important to point out that the real word frequency of these authorized indications is very low: only 6.17 and 1.23% of the enrolled patients in our study suffered from bipolar disorder and ADHD respectively, and none was diagnosed with schizophrenia in our study. A possible interpretation of this finding could be that generally for adolescents with bipolar disorder or ADHD an inpatient care is needed only when psychiatric emergency presents, like severe mood disorder of psychosis, and in this case the main diagnosis reported are these.

The fact that mood disorders and eating disorders do not have a specific treatment in adolescence is confirmed by the high frequency of off label prescriptions, in our series, among subjects with these disorders.

Another consideration must be done, concerning the specificity of the adolescent age and psychiatric diagnosis: this age represents a particular phase for neurodevelopmental processes (Pablo et al., 2019) when many symptoms can appear, even intense or severe, without this meaning a definite psychiatric diagnosis. It can be difficult to diagnose a psychotic/bipolar/schizophrenic disorder at this age, when many maturational processes are still in progress. Moreover, clinical pictures cannot be clear at the first hospitalization taking place during a psychiatric emergency. The burden of the potentially stigmatizing power of a psychiatric diagnosis can make it difficult to formalize it, mainly in the stormy context of the emergency hospitalization. This all factors can contribute to the evidence of a relatively low frequency of diagnosis of psychosis and to the use of antipsychotics out of indication, resulting in an off-label prescription.

Concerning the field of neurological disorders, off-label prescription is mainly needed in drug-resistant epilepsies, so when there is no therapeutic alternative (other therapeutic options have been tried).

Most of the identified ADRs affect the nervous system: it is critical to consider the dynamic effect of antipsychotic and antiepileptic drugs on the immature brain, which demonstrates plasticity in its ability to adapt to the external milieu and preventative interventions. In this study half of the extrapyramidal effects were determined by first generation antipsychotics and the other half by second generation antipsychotics, although the latter should be administered less frequently (Stahl 2017). Clinicians prescribing these medications should familiarize themselves with the most common adverse events, and work with pharmacists and pharmacologists to recognize them early through monitoring (neurological examination but also electrocardiograms, absolute neutrophil counts, blood glucose, blood LDL and weight).

In our study the overall incidence of therapeutic inappropriateness was 5.8%, consistent with the literature data (Lewis et al., 2009; Alshehri et al., 2017); 82% of them were associated with drug-drug interactions (mainly due to contraindicated drug combinations) and 29% of them led to overdose. 18% of the identified ADRs was preventable and correlated to therapeutic inappropriateness, a percentage which is consistent with the results from 20 studies which we reviewed in a systematic manner. From our results, therapeutic inappropriateness discovered to be a very high-risk factor for having an ADR (OR 6.69; IC95% 3.03–14.77; *p* = 0.000).

Furthermore, 73% of therapeutic inappropriateness were linked to psychotropic drugs prescription (Clothiapine, Clozapine, Haloperidol, Lithium, Olanzapine, Quetiapine Risperidone) and 27% were linked to antiepileptic prescriptions (Phenytoin, Primidone, Valproic Acid). The important issue to focus on is therapeutic inappropriateness consisting in drug-drug interactions and improper uses. A total of 113 drug-drug interactions were found and 27% of patients in our study experienced a drug-drug interactions. Valproic Acid caused 23 (20%) drug-drug interactions and appeared to be a broad-spectrum enzyme inhibitor as it inhibits the activity of UGT enzyme (UGT1A4 and UGT2B7) as well as CYP2C9 and, weakly, CYP2E1. Valproic Acid increases the serum concentration of Phenobarbital, Lamotrigine and Ethosuximide, resulting in possible enhancement of the adverse/toxic effect of its substrate. Valproate Products may decrease the protein binding of Fosphenytoin-Phenytoin: this appears to lead to an initial increase in the percentage of unbound (free) phenytoin (Perucca 2006; Zaccara and Perucca 2014).

Also, Carbamazepine and Phenobarbital caused drug-drug interactions (N = 26, 23%) both inducing CYP450 and glucuronyl transferase enzyme and so reducing serum concentration of substrate of the same enzyme. In our study we detected carbamazepine interactions with phenytoin, rufinamide, valproic acid, phenobarbital, levetiracetam, desmopressin, clobazam and levothyroxine.

We observed a poorly described particular drug–drug interaction that caused significant extrapyramidal adverse reaction: the concomitant use of lithium with first/second generation of antipsychotic. Considering that the extrapyramidal side effects are due an imbalance between dopaminergic and cholinergic systems, and considering the fact that lithium and psychotropic drugs are known to decrease the amount of dopamine, it is plausible to consider it as a pharmacodynamic drug-drug interaction (Baastrup et al., 1976; Addonizio 1985; Sachdev 1986; Tuglu et al., 2005).

This is the first detailed analysis of off-label prescription patterns in Child Neuropsychiatry in Italy. The context of Italian Child Neuropsychiatry, which unlike other European realities deals with both neurological and psychiatric pathology in the pediatric age, offers the possibility of a unique perspective that allows to extend the analysis of the problem of off-label prescribing to a wide spectrum of clinical areas, both very relevant: epilepsy, that is among the most frequent neurological pathologies in children, often due to rare diseases (Amann et al., 2013) and psychiatric disorders; both have very little evidence for drug use.

The study has several limitations. The first limitation is the retrospective nature of the study and its structure that do not allow the detection of variables with a potential confounding role. In addition, the low number of events with which some outcome occurred in our study did not allow the execution of appropriate subgroup analyzes in order to explore the validity of the relationships between different variables. Nevertheless, retrospective studies are an important tool to study rare diseases and findings of this study can form the basis on which prospective studies are planned.

## Conclusion

This study assessed the prescriptive and safety profiles of pharmacological treatment for the inpatient care of children and adolescents with neuropsychiatric disorders. The findings of a high prevalence of off-label prescription highlights unmet needs in pediatric neuropsychopharmacology, mainly for what in psychiatry is concerned. This study has not detected greater risks associated with off-label prescriptions supporting the relevance of research in this field. As members of a multidisciplinary team, pharmacists and pharmacologists may collaborate with clinician providing them their expertise by medication review of pharmacological therapies, leading to increased therapeutic appropriateness.

These first results give us several insights into areas for improvement, mainly preventing prescribing inappropriateness and polypharmacy. This study has shown that to increase therapeutic safety of neuropsychiatric therapies in pediatrics, monitoring of drug interactions and therapeutic appropriateness are needed both in on- and off-label use of drugs. Multidisciplinary team should take any effort to optimize the appropriateness and the safety of therapies as well as the resources of the National and Regional Health Systems.

## Data Availability

The raw data supporting the conclusions of this article will be made available by the authors, without undue reservation.
